# Mobile Phone Addiction as an Emerging Behavioral Form of Addiction Among Adolescents in India

**DOI:** 10.7759/cureus.23798

**Published:** 2022-04-04

**Authors:** Navya Gangadharan, Amod L Borle, Saurav Basu

**Affiliations:** 1 Community Medicine, Maulana Azad Medical College, New Delhi, IND; 2 Epidemiology and Public Health, Indian Institute of Public Health, Public Health Foundation of India, New Delhi, IND

**Keywords:** behavioral addiction, mobile phone addiction, dependence, adolescent health, smartphone addiction

## Abstract

Introduction: The advancements in mobile phones from simple basic phones to featured phones and smartphones resulted in the penetration of technology to different groups of people irrespective of age, gender, or region. Thus, mobile phone addiction has evolved as a form of behavioral addiction found to be increasingly prevalent among adolescents too. The study aimed to determine the prevalence of mobile phone addiction among adolescents and its associated risk factors among adolescents.

Method and results: A community-based, cross-sectional study was conducted among 264 adolescents (10-19 years) of low-income urban areas of Delhi. The prevalence of mobile phone addiction in the participants was 33.0% (95% CI: 27.2-38.6). The addiction was higher among boys (33.6%) than girls (32.3%) (p=0.835). Mobile phone addiction was found to be significantly higher among those adolescents who had ≥3 siblings, those belonging to nuclear families, and among late-onset users (≥16 years). Late-onset users (adjusted odds ratio {aOR}: 3.398; 95% CI: 1.307-8.833) and ≥3 siblings (aOR: 1.980; 95% CI: 1.141-3.437) were independent predictors of mobile phone addiction. The mean time spent on mobile phones was significantly higher among those with addiction but no significant gender difference was found between time spent on phones and addiction.

Conclusion: The high prevalence of mobile phone addiction found in our study is an indication of the potential public health concern posed by mobile phone use among adolescents in urban settings. Hence, it is essential to limit the access to mobile phones for important utility purposes for adolescents.

## Introduction

Mobile phones, in the last decade, have evolved from a primary tool of interpersonal communication to that facilitating group communication, and such exponential transformation was seen further with the introduction of smartphones [1]. The advancements in mobile phones from simple basic phones to featured phones and smartphones resulted in the penetration of technology to different groups of people irrespective of age, gender, or region. The major advantage is that it is portable and real-time with the availability of Internet anywhere, anytime with features of browsing and wide range of social media applications. These features have resulted in greater acceptance and higher usage of smartphones not only among adults but also among children and adolescents.

In recent years, smartphones evolved to be multitasking and have displaced electronic devices such as a computer, camera, and many others which have made us to use them more often [2]. Excessive use of such technologies may put one at the risk of adverse effects such as isolation and feeling of loneliness, decreased interpersonal relationships, and social interactions in them [3,4].

Globally, the prevalence of mobile phone addiction is varying from 2.4% to as high as 60.3% among adolescents and school-going children [2,5]. India is one of the largest and fastest-growing markets for digital consumers, with 560 million Internet subscribers (nearly 41%) in 2018, second only to China. The average Indian social media user spends 17 hours on the platforms each week, more than social media users in China and the United States. Half of India’s entry-level users for smartphones are between 15 and 24 years old and mostly students [6]. Chat, video streaming, browsing, social networking, and image apps are the most engaging and account for more than 50% of the total time spent on smartphones. India has the highest number of adolescents (253 million) and they constitute one-fifth of the Indian population and 22% of them live in urban areas [7]. In this context, the present study was conducted with the objective of determining the prevalence and predictors of mobile phone addiction among adolescents in low-income urban areas of Delhi.

## Materials and methods

A community-based, cross-sectional study was conducted from October 2020 to March 2021 in two low-income urban agglomerates in the North East and Central Delhi districts of Delhi which represent the catchment areas of the community medicine department of a medical college in New Delhi. The ethical clearance was obtained for the study from the Institutional Ethics Committee, Maulana Azad Medical College, New Delhi (#F1/IEC/MAMC/73/01/2020/NO 26).

The sample size was estimated using the formula 4PQ/D2, where P was the prevalence of mobile phone dependence obtained from a study done by Nikhita et al. in Navi Mumbai as 31%, and absolute error as 6% [8]. Thus, the sample size calculated came out to be 228 and after adjusting for 10% non-response rate, the calculated sample size was 250.

The study instrument was an interview schedule that was prepared by the investigator after doing pretesting in a sample of 10 adolescents. The interview schedule covered details about the bio-data and socio-demographic characteristics. Mobile phone addiction was assessed using the previously validated Mobile Phone Addiction Scale (MPAS) by Basu et al. consisting of 20 items with each measured on a six-point Likert scale [9]. The 20 items covered six domains of substance dependence which are intense desire, impaired control, harmful use, withdrawal, tolerance, and decreased interest in alternate pleasures as per the International Classification of Diseases (ICD)-10 classification of mental and behavioral disorders: clinical descriptions and diagnostic guidelines [10]. The original MPAS had revealed a four-factor structure on principal component analysis. Each domain was diagnosed as positive when 50% or more items had positive responses (four and more on the Likert scale). Mobile phone addiction was diagnosed when three or more domains were positive in a respondent. The MPAS English was linguistically validated into the local language Hindi, through a standardized forward and backward translation method, by two professionals who had native fluency in both languages. The steps included forward translation of MPAS English into Hindi by the first professional, backward translation of Hindi translation into English by the second professional, comparison of the original MPAS English and backward translated English version, and repetition of the process until there was adequate matching of both the versions. The reliability assessment of this MPAS Hindi instrument observed a Cronbach's alpha of 0.841 which was suggestive of good reliability. No increase of >0.1 in the Cronbach's alpha was obtained when items were removed one by one. Inspection of the item-total correlation showed a range of 0.06-0.622 with all except one correlation coefficient (item one) <0.3.

In each area, households were selected by systematic random sampling. The line list of households in each block was obtained with the help of frontline health workers (Accredited Social Health Activists (ASHAs) and Anganwadi workers). There was a total of 3201 households in the study area and a sampling interval was calculated as 12. Then standing at the first house in A block, the first household was selected by simple random sampling. A lot was taken by lottery method for selecting the first household and thereafter every 12th house was selected for the study. Similarly, it was done for all other blocks till the sample size was reached. From the selected household, adolescent between the age of 10 and 19 years was selected for interview. If there were more than one adolescent meeting the inclusion criteria in the selected household, then the study participant was selected through simple random sampling using the lottery method. If the participant was not present on the day of visit, they were covered in two subsequent visits. If a particular house was found locked on three consecutive visits, then the eligible study subject was dropped from the study, and the next eligible participant was chosen. In case no eligible person was found then next systematic house was taken.

The data collected were entered in Microsoft Excel and analyzed using SPSS version 25 (Armonk, NY: IBM Corp.). The quantitative data were expressed as mean and standard deviation or median and interquartile range and categorical data as frequencies and percentages. Chi-square test or Fisher's exact test was used to assess the difference between proportions. Student's t-test and Mann-Whitney U test were used to assess the difference in distribution of quantitative variables which followed normal distribution/non-normal distribution. Binomial logistic regression was done to identify the independent predictors for mobile phone addiction. A p-value less than 0.05 was taken as significant.

## Results

The study was conducted among 264 adolescents. The response rate was 93.8% among boys and 94.6% among girls. A total of 122 (46.2%) boys and 142 (53.8%) girls were enrolled in the study. The mean age of adolescents was 14.2 ± 2.4 years of which the mean age of boys was 14.4 ± 2.3 years and that of girls was 14.1 ± 2.4 years. The sociodemographic characteristic of the adolescents is shown in Table 1.

**Table 1:** Sociodemographic characteristics of the adolescents, n=264. SD: Standard deviation

Sociodemographic characteristics		Frequency	Percentage
Age	10-13 years	110	41.6
	14-17 years	124	47.0
	≥18 years	30	11.4
Gender	Boys	122	46.2
	Girls	142	53.8
Socioeconomic status	I Upper	1	0.4
	II Upper middle	51	19.3
	III Middle	115	43.6
	IV Lower middle	90	34.1
	V Lower	7	2.7
Number of siblings	None	11	4.2
	1	50	18.9
	2	117	44.3
	≥3	86	32.6
Number of family members	≤4	50	18.9
	5-7	204	77.3
	≥8	10	3.8
Education status	Primary school	7	2.7
	Middle school	113	42.8
	High school	70	26.5
	Higher secondary and above	74	28.0
Working status of parents	None of them working	10	3.8
	One of them working	197	74.6
	Both are working	57	21.6
Type of family	Nuclear	181	68.6
	Joint	83	31.4
Type of mobile phone used regularly	Smartphone	258	97.7
	Basic phone	6	2.3
Access to mobile phone	Own a mobile phone	85	32.2
	Borrowed from parents	167	63.3
	Borrowed from siblings	7	2.7
	Borrowed from friends	5	1.9
Age of first use	<10 years	76	28.8
	11-15 years	168	63.6
	>16 years	20	7.6
Purpose of mobile phone use (multiple responses)	Gaming - online/offline	151	57.2
	Social networking - WhatsApp/Instagram, etc.	210	79.5
	Watching videos on YouTube/similar apps	230	65.2
	Chatting/video calling	172	87.1
	Others	13	4.9
Use of Internet on mobile phone	Yes	259	98.1
	No	5	1.9
Access to Internet	Wi-Fi	87	32.9
	Cellular data	126	47.7
	Both	46	17.4
	Not using any	5	1.9
Internet usage while using mobile phone	Very often	177	68.3
	Less often	82	31.7
Preference of activity	Prefer outdoor activities like playing games	68	25.8
	Prefer using mobile phone and staying indoors	196	74.2
Skip food due to phone use in past seven days	Yes	16	6.1
	No	248	93.9
Time spent on mobile phone/day (mean ± SD)	Weekdays	2.9 ± 1.5 hours	
	Weekends	3.8 ± 1.2 hours	

The prevalence of mobile phone addiction was found to be 33.0% (95% CI: 27.2-38.6) and was more among boys (33.6%) than girls (32.3%) but not significant statistically. The most commonly met ICD-10 diagnostic criteria were tolerance and withdrawal in this population. Tolerance to use mobile phones was found to have higher prevalence among adolescents (54.5%) followed by withdrawal to its use (47%). Within each domain, it was observed that girls predominated more than boys (Table 2). It was also observed that for almost all items girls responded more positively than boys except for Q1, 9, 19. Girls had an intense desire to check WhatsApp/Facebook even while resting (16.2%) and it was found to be significantly more than boys (6.6%) (p=0.015) (Table 3).

**Table 2:** Distribution of positive domains of MPAS among adolescents, n=264. MPAS: Mobile Phone Addiction Scale

Item no.	Domain	Total, n (%)	Boys, n (%)	Girls, n (%)
1	Intense desire	21 (8%)	9 (42.5)	12 (57.5)
2	Impaired control	67 (25.4)	33 (49.3)	34 (50.7)
3	Withdrawal	124 (47%)	54 (43.5)	70 (56.5)
4	Tolerance	144 (54.5)	69 (47.9)	75 (52.1)
5	Decreased interest in alternate pleasures	71 (26.9)	33 (46.5)	38(53.5)
6	Harmful Use	66 (25.0)	30 (45.5)	36 (54.5)
Prevalence of mobile phone addiction in the study (adolescents having ≥3 positive domains)		87 (33)	41 (33.6)	46 (32.3)

**Table 3:** Distribution of positive responses to MPAS among adolescents, n=264. MPAS: Mobile Phone Addiction Scale Source: Basu et al. (2018) [9].

S. no.	Statement	Positive responses			
		Total, n (%)	Boy (n=122), n (%)	Girl (n=142), n (%)	χ^2^, df, p-value
1	Usually check your WhatsApp/SMS/Facebook notifications as soon as you receive them during the day	88 (33.3)	42 (34.4)	46 (32.4)	0.122, 1, 0.727
2	Usually check WhatsApp/SMS/Facebook notifications received even while resting/in light sleep	31 (11.7)	8 (6.6)	23 (16.2)	5.884, 1, 0.015
3	Usually impulsively check for WhatsApp/SMS/Facebook notifications while attending classes or studying at home	29 (11.0)	12 (9.8)	17 (12.0)	0.306, 1, 0.580
4	Usually check your mobile phone for messages/gaming/surfing even while attending classes	35 (13.3)	14 (11.5)	21 (14.8)	0.626, 1, 0.429
5	Usually check your mobile phone for new messages or notifications right after waking up from sleep	57 (21.6)	25 (20.5)	32 (22.5)	0.162, 1, 0.687
6	Constantly checking my smartphone so as not to miss conversations between my friends/other people on Twitter/Facebook/WhatsApp	38 (14.4)	12 (9.8)	26 (18.3)	3.824, 1, 0.051
7	Having a hard time concentrating in class, while doing assignments, or while working due to mobile use	45 (17.0)	20 (16.4)	25 (17.6)	0.068, 1, 0.794
8	Preferring talking with my smartphone buddies to hanging out with my real-life friends or with the other members of my family	110 (41.7)	49 (40.2)	61 (43.0)	0.211, 1, 0.646
9	Usually check your mobile phone even while engaged in group participation	104 (39.4)	49 (40.2)	55 (38.7)	0.056, 1, 0.812
10	Using your mobile phone longer than you had intended to Using your mobile phone longer than you had intended to	160 (60.6)	72 (59.0)	88 (62.0)	0.240, 1, 0.624
11	Always thinking that you should shorten your mobile phone usage time	127 (48.1)	55 (45.1)	72 (50.7)	0.831, 1, 0.362
12	The people around you complain that you don’t pay attention to them due to mobile phone use	47 (17.8)	20 (16.4)	27 (19.0)	0.308, 1, 0.579
13	Get annoyed or shout if someone asks you to decrease the use of mobile phone	73 (27.7)	32 (26.2)	41 (28.9)	0.229, 1, 0.632
14	Feeling impatient and fretful when you are not holding your smartphone	95 (36.0)	40 (32.8)	55 (38.7)	1.007, 1, 0.316
15	Experience stress when not using your mobile phone	75 (28.4)	38 (32.1)	37 (26.1)	0.836, 1, 0.360
16	Experiencing lightheadedness or blurred vision due to excessive smartphone use	95 (36.0)	37 (30.3)	58 (40.8)	3.151, 1, 0.076
17	Feeling pain in the wrists or at the back of the neck while using a smartphone	85 (32.2)	37 (30.3)	48 (33.8)	0.363, 1, 0.547
18	Feeling tired and lacking adequate sleep due to excessive smartphone use	107 (40.5)	49 (40.2)	58 (40.8)	0.013, 1, 0.911
19	Cannot imagine living without my mobile phone	129 (48.9)	61 (50.0)	68 (47.9)	0.117, 1, 0.732
20	Do you compulsively respond to calls/messages at places where it is dangerous to do so (driving/crossing the road)	16 (6.1)	5 (4.1)	11 (7.7)	1.534, 1, 0.216

The association of various predictors with mobile phone addiction is shown in Table 4 and age of first use (>16 years), nuclear family, and ≥3 siblings were significantly found to be associated with mobile phone addiction. A binary logistic regression yielded age of first use (16 years) (adjusted odds ratio {aOR}: 3.398; 95% CI: 1.307-8.833) (p=0.012) and ≥3 siblings (aOR: 1.980; 95% CI: 1.141-3.437) (p=0.015) as independent predictors of mobile phone addiction (Table 5).

**Table 4:** Association of factors associated with mobile phone addiction among adolescents, n=264.

Factors		Mobile phone addiction		Total	p-Value
		Present n (%)	Absent n (%)		
Age category	<15 years	40 (28.8)	99 (71.2)	139 (100.0)	0.128
	≥15 years	47 (37.6)	78 (62.4)	125 (100.0)	
Gender	Boys	41 (33.6)	81 (66.4)	122 (100.0)	0.835
	Girls	46 (32.4)	96 (67.6)	142 (100.0)	
Socioeconomic status of the family	Upper class	17 (32.7)	35 (67.3)	52 (100.0)	0.959
	Middle class	37 (32.2)	78 (67.8)	115 (100.0)	
	Lower class	33 (34.0)	64 (66.0)	97 (100.0)	
No of siblings	None	0 (0.0)	11 (100.0)	11 (100.0)	0.018
	1	15 (30.0)	35 (70.0)	50 (100.0)	
	2	35 (29.9)	82 (70.1)	117 (100.0)	
	≥3	37 (43.0)	49 (57.0)	86 (100.0)	
Number of family members	≤4	13 (26.0)	37 (74.0)	50 (100.0)	0.478
	5-7	71 (34.8)	133 (65.2)	204 (100.0)	
	≥8	3 (30.0)	7 (70.0)	10 (100.0)	
Education of the adolescents	Up to middle school	35 (29.2)	85 (70.8)	120 (100.0)	0.249
	High school	22 (31.4)	48 (68.6)	70 (100.0)	
	Higher secondary and above	30 (40.5)	44 (59.5)	74 (100.0)	
Parent’s working status	One parent working	65 (33.0)	132 (67.0)	197 (100.0)	0.457
	Both working	17 (29.8)	40 (70.2)	57 (100.0)	
	None of them working	5 (50.0)	5 (50.0)	10 (100.0)	
Type of family	Nuclear	67 (37.0)	114 (63.0)	181 (100.0)	0.038
	Joint	20 (24.1)	63 (75.9)	83 (100.0)	
Type of mobile phone used	Smartphone	84 (32.6)	174 (67.4)	258 (100.0)	0.399
	Basic phone	3 (50.0)	3 (50.0)	6 (100.0)	
Access to phone	Own a mobile phone	27 (31.8)	58 (68.2)	85 (100.0)	0.777
	Borrowed from others	60 (33.5)	119 (66.5)	479 (100.0)	
Age of first mobile phone use	<10 years	19 (25.0)	57 (75.0)	76 (100.0)	0.012
	11-15 years	56 (33.3)	112 (66.7)	168 (100.0)	
	≥16 years	12 (60.0)	8 (40.0)	20 (100.0)	
Use of Internet	Yes	84 (32.4)	175 (67.6)	259 (100.0)	0.335
	No	3 (60.0)	2 (40.0)	5 (100.0)	
Access to Internet	Cellular data only	30 (34.5)	57 (65.5)	85 (100.0)	0.506
	Wi-Fi only	38 (30.2)	88 (69.8)	126 (100.0)	

**Table 5:** Multivariate logistic regression of independent predictors for mobile phone addiction. OR: odds ratio; aOR: adjusted odds ratio, 95%; CI: confidence interval, 95%

Variables	Exposure level	Crude OR (95% CI)	aOR (95% CI)	p-Value
Number of siblings	≥3	1.933 (1.129-3.309)	1.980 (1.141-3.437)	0.015
	<2	1		
Type of family	Nuclear	1.897 (1.056-3.409)	1.703 (0.935-3.103)	0.082
	Joint	1		
Age of first mobile use	≥16	3.380 (1.327-8.610)	3.398 (1.307-8.833)	0.012
	<16	1		

A statistically significant difference in the mean time spent on weekdays and weekends was observed among those with mobile phone addiction. On working days boys spent a mean time of 3.0 ± 1.5 hours/day whereas girls spent 2.9 ± 1.6 hours/day. On weekends boys spent 3.9 ± 1.0 hours/day whereas girls spent 3.9 ± 1.3 hours/day, but there was no statistically significant difference between gender and time spent on phone. It was also observed that more time was spent on weekends compared to working days on mobile phones by girls and boys.

## Discussion

The prevalence of mobile phone addiction was found to be 33% in the present study among adolescents. Similar studies conducted in India earlier had reported prevalence of MPD as 31.3% by Nikhita et al. in Navi Mumbai and 30.3% in Haryana by Jamir et al. [8,11]. So, our findings are consistent with earlier studies done in India. A slightly higher prevalence of addiction-like behavior was proposed by Basu et al. (40%) in Delhi but this difference could be because of the different study populations (medical students) in which the study had been conducted [9]. Medical students may be at higher levels of stress and burnout which could render them at risk for developing addictions more than the general population.

When compared with other Global studies the prevalence of mobile phone addiction has ranged from 2.4% to 60.3% [2,5]. Studies done in South Korea by Cha et al. and in China by Chen et al. had shown prevalence similar to this study (30.9% and 29.8%) though they used scales different from this study [12,13]. This emphasizes that our prevalence could be compared with other nations especially South East Asia region. Whereas some studies like Shi et al., in 2021, in China (41.2%) had reported a higher prevalence which could be because of heterogeneous scale (MPAI), and the study focussed on college students [14]. Similarly, Alsalameh et al. in Saudi Arabia who enrolled an older population (19-32 years) also had reported a higher prevalence than our study [2]. The differences in prevalence estimated in other studies done in the United Kingdom (10%), Spain (14.8%), Italy (6.3%), and Iran (17.7%) could be because of different scales used for estimation of mobile phone addiction and different study population (mainly school going students who have limited access to mobile phone during school times) [15-17]. The present study was conducted during the pandemic of COVID-19 when nationwide lockdowns were imposed, hence the increased accessibility of mobile phones for online academics would have resulted in higher prevalence too. Along with that, the rapid changes in the use of Internet evolving in recent years could also have led to increased prevalence among adolescents.

In our study, mobile phone addiction was more prevalent among late adolescents (>15 years). Whereas, Gallimberti et al. in their study in Italy among adolescents of age 11 to 13 years reported that problematic use of phones for text messaging increased with age, especially among girls [18]. Similarly, Lopez-Fernandez et al. in their study done among British adolescents had reported a higher prevalence of problematic mobile phone use among early adolescents (11-14 years) [15]. As our study did not just pertain to text messaging and covered all aspects of mobile phone use, this variation can be considered. Also, differences in family background and urbanization might also have resulted in the early adolescents being more addicts in previous studies.

A higher prevalence of addiction was seen among boys (33.6%) than girls (32.4%) in our study but was not statistically significant. The higher prevalence among boys was also observed in other studies done by Zou et al. (23.2% vs 22.3%), Chen et al. (30.3% and 29.3%), Basu et al. (41.2% vs 38%), Nikhita et al. (OR 1.91 {95% CI 1.23-2.99}) and Jamir et al. (OR 2.82 {95% CI 1.43-5.59}) [8,9,11,13,19]. In contradiction, many studies have found that female gender is a risk factor for mobile phone addiction [14,20,21]. The higher prevalence among boys in our study could be because of more recreational time that they have at their homes compared to girls who often get involved in house chores as usually seen in Indian families.

In the present study, a significant association was found between mobile phone addiction and number of siblings. It was observed that mobile phone addiction was higher among adolescents with more than one sibling than among those who were single child. Similarly, it was more prevalent among families who had a higher number of family members (≥5 members) but the findings were not significant. Similar findings were found in a study by Jamir et al. in Haryana but no significant relation was found [11]. This could be because higher the number of siblings or members in the family, higher would be the engagement of other media like television and computers to them. This could potentially let the adolescents in such families to depend on mobile phones during their leisure time.

Our study showed a significant association between family type and mobile phone addiction (p=0.038). The addiction was observed to be more in nuclear families (37%) than in joint families (24.1%). Similar findings were also reported by Nikitha et al. and a significant association was found [8]. This could be attributed to the limited supervision by parents of their children when they are working in nuclear families.

Late ages of initiation of mobile phone usage (≥16 years) were found to have significantly higher proportion of mobile phone addiction than earlier ages. In late adolescence period (≥16 years) they would be influenced by peer groups and curiosity would be more which result in excessive indulgence in such activities. Whereas with an early age of initiation of mobile phone use they would be in alignment with its use which minimizes the risk for addiction.

The mean time spent on mobile phones was significantly higher among adolescents with mobile phone addiction on all days (p=0.011). Also, it was observed that on weekends the overall time spent was higher than other days in our study (p=0.001). But no significant difference was observed in time spent by boys and girls. Cha et al. in their study had also reported that risk group used smartphones longer than normal users [12]. Nikitha et al. had also observed that increasing amount of time spent on mobile phones per day was significantly associated with mobile phone dependence [8].

## Conclusions

A major limitation of the study was no baseline data were available about the addiction behavior to compare whether the present COVID-19 pandemic had resulted in increased prevalence in this population. Also, as it was a self-reported survey, underestimation of actual time of mobile phone use might have happened. In the future technological records of smartphone use should also be investigated along with self-reporting as it will provide a comprehensive explanation of actual smartphone usage patterns. As it was a cross-sectional study the temporality of the predictors could not be established.

To conclude, the high prevalence of mobile phone addiction found in our study is an indication of the potential public health concern posed by mobile phone use among adolescents in urban settings. Adolescents who are at a transition stage are at a higher risk for technological dependence and addictions nowadays. Hence, it is essential to limit the access to mobile phones for important utility purposes to adolescents. Studies focusing on increasing burden of technology addiction and its effect on psychosocial and physical growth of adolescents is an area to ponder in the future.
